# Effect of Diversity Education on Young Adolescents in Japan: Toward the “Do No Harm” Principle

**DOI:** 10.3390/ijerph20064900

**Published:** 2023-03-10

**Authors:** Takashi Izutsu, Shodai Sunagozaka, Yuhei Yamada, Atsuro Tsutsumi

**Affiliations:** 1 Graduate School of Arts and Sciences, The University of Tokyo, Tokyo 153-8902, Japan; 2 Graduate School of Human and Socio-Environmental Studies, Kanazawa University, Kanazawa 920-1192, Japan; 3 Porque, The Organization of Persons with Psychosocial Disabilities, Tokyo 143-0026, Japan; 4 Institute of Transdisciplinary Sciences for Innovation, Kanazawa University, Kanazawa 920-1192, Japan

**Keywords:** education program, diversity, disability, human rights, inclusion, mental health, adolescence

## Abstract

This study evaluated the impact of a semi-structured diversity education program on young adolescents, which included five 45-min sessions facilitated by schoolteachers using an instructors’ manual. The study compared changes in knowledge and attitude related to diversity, self-esteem, and mental health among participants before and after the program. The participants were 776 junior high school students. Self-esteem and mental health conditions were assessed with the Rosenberg Self-Esteem Scale (RSES) and Kessler 6-Item Psychological Distress Scale (K6). The ratio of those who answered the knowledge and attitude questions correctly increased significantly for most questions, while the ratio decreased significantly for two questions. The RSES scores improved significantly after the program, but the difference was very small. Mental health, as measured by K6, became significantly worse after the program. A logistic regression analysis indicated that lower K6 scores before the program and worse academic grades had significantly higher odds ratios; being a girl, not having a disability, and having close friends were associated with worse K6 scores after the program. Further, this indicates the importance of developing processes based on evidence and the “nothing about us without us” principle.

## 1. Introduction

Diversity and inclusion have become global priorities in various international agreements. In particular, the United Nations 2030 Agenda for Sustainable Development and the Sustainable Development Goals have adopted the “leave no one behind” and “reach the furthest behind first” as foundational principles and made diversity and inclusion one of the top global priorities [1]. Furthermore, the international community has developed a human rights system through conventions and follow-up mechanisms, including the Convention on the Elimination of All Forms of Racial Discrimination [2], the Convention on the Elimination of All Forms of Discrimination against Women [3], and the Convention on the Rights of Persons with Disabilities [4]. These conventions promote the development of national systems to protect and promote diversity and inclusion.

Education plays a key role in increasing individual and societal awareness and nurturing inclusive cultures and systems to protect and promote diversity and inclusion. Research has been conducted with various foci to examine the effectiveness of diversity education. For example, early studies (from the 1950s–60s) examined school-based educational interventions to improve attitudes toward race [5,6]. A meta-analysis of 81 studies included 122 intervention–control comparisons of structured programs to reduce prejudice or promote positive intergroup attitudes among children and adolescents. This meta-analysis showed that interventions based on direct contact experiences, taken together with social-cognitive training to promote empathy and perspective-taking, showed the strongest effect sizes; the effects varied based on social status, target group, and type of outcome assessment [7]. Regarding gender and sexual diversity, according to a meta-analysis, education, contact, and the combination of education and contact had a medium-sized effect on sexual prejudice. This indicated the possible power of using entertainment media to promote tolerance [8]. Furthermore, experiential learning in a board game format was found to improve knowledge in a non-threatening and lasting way compared to knowledge-centered and non-experiential learning modalities [9]. However, in contrast, Kroneman et al. [10] indicated that a school-based peer-educator intervention on attitudes toward sexual minorities had limited effects.

There have also been studies on educational interventions related to disabilities. A meta-analysis was conducted on the effects of 20 school-based interventions to improve disability awareness and attitudes from kindergarten to secondary school in the Republic of Korea. The study found that contact-based interventions, use of materials, role-playing, and human rights interventions were effective factors in achieving such improvement [11]. In addition, a study targeting secondary school students demonstrated that simulation and modeling to put participants in place of persons with disabilities was more effective than other countermeasures, such as providing knowledge through lectures or experiencing sports for persons with disabilities. These modalities were effective in improving the students’ attitudes [12]. 

However, few studies with strong designs and large sample sizes have examined educational interventions to reduce the stigma against persons with mental health conditions or psychosocial disabilities. A narrative review [13] found that such interventions lead to a consistent pattern of short-term benefits of positive attitude change and knowledge improvement (with less evidence). However, the knowledge of their long-term effects remains limited. Further, these programs do not adequately consider the perspectives of persons with mental health conditions or psychosocial disabilities and have a limited focus on behavioral change. Social contact was the most effective type of intervention in improving stigma-related knowledge and attitudes. Holzinger et al. [14] reviewed 51 studies, more than half of which were conducted among school students. Most of these studies reported positive intervention effects, especially in improving knowledge compared with reducing the tendency to distance oneself from persons with mental health conditions or psychosocial disabilities. However, the long-term effects of most of these interventions remain unknown. A combination of education and contact with persons with mental health conditions or psychosocial disabilities was more effective than education alone. Further, a study conducted in UK secondary schools indicated that two workshops produced positive changes in participants’ attitudes toward persons with mental health conditions or psychosocial disabilities. The impact was higher among female students and students in contact with persons with mental health conditions or psychosocial disabilities [6].

A limited number of studies on educational interventions to ensure the diversity and inclusion of students have been reported in Japan. A study targeting medical university students indicated that a one-hour educational program demonstrated positive attitudinal changes related to distance from persons with mental health conditions or psychosocial disabilities, approaches to psychiatric services, and perspectives on human rights, including the importance of independence in social life [15]. However, to the best of our knowledge, the effect on the mental health of those who undergo educational programs regarding diversity and inclusion themselves has not been examined in Japan or even globally.

Concepts and terminologies related to diversity and inclusion evolve over time. Taking the rights of persons with disabilities as an example, models have changed from the charity model, which considers persons with disabilities as vulnerable objects of charity, to a medical model that often views disability only from a medical lens, such as requiring prevention and treatment. The current social model is based on human rights and views environmental, institutional, and attitudinal barriers as key factors and targets for interventions related to disability [16]. In the social model, persons with disabilities are agents of change rather than passive receivers of determined support and are expected to participate in relevant decision-making processes in a meaningful way. The terms have also changed from “handicapped people” or “disabled people” to “persons with disabilities.” These concepts were changed based on discussions with organizations of persons with disabilities, good practices, lessons learned, evidence, and cultural contexts. This means that diversity and inclusion education requires constant updates through co-production, and culture, and context sensitivity. In particular, the current ethical standards underscore the importance of evidence-based interventions that involve key stakeholders in planning, implementation, and evaluation based on the principles of “do no harm” and “nothing about us without us.” There is increasing awareness that without such considerations, educational programs could harm people and society. Nevertheless, in the field of diversity and inclusion education, some programs have been developed with “good intentions” but without systematically evaluating whether the content is updated, evidence-based, safe, and co-created with key stakeholders, including women, persons with disabilities, and other relevant populations.

This study evaluated a semi-structured education program on diversity and inclusion in four cities in Japan. The program was developed based on experience and good intentions but with limited reference to evidence, new concepts and terminologies, and the voices of stakeholders with diverse backgrounds.

## 2. Methods

### 2.1. Participants

A total of 776 students from four junior high schools in the four suburban cities of Japan participated in the education program. Study participants who responded to a question on gender included 210 students from city A (boys: 108 (51.4%), girls: 95 (45.2%), others: 7 (3.3%); age range: 12–14 years; mean [M]: 13.29 years, standard deviation [SD]: 0.59), 99 students from city B (boys: 50 (50.5%), girls: 49 (49.5%); age range: 12–14 years; M: 12.86 years, SD: 0.38), 129 students from city C (boys: 75 (58.1%), girls: 52 (40.3%), others: 2 (1.6%); age range: 12–13 years; M: 12.82 years, SD: 0.38), and 235 students from city D (boys: 129 (54.9%), girls: 106 (45.1%); age range: 12–13 years; M: 12.66 years, SD: 0.48). A total of four junior high schools, one from every four cities, consisting of seven classes from the school in city A, four in city B, four in city C, and 11 in city D, participated in the study. A teacher from each class presented the educational program to each class. In other words, the teachers who implemented the program differed from class to class but based on an instruction guide. The vendor who developed this program requested the participation of schools with which it had prior relationships; four schools agreed to participate in this study. 

### 2.2. Instruments and Measures

*Demographic Data*

Demographic data, including age, gender, academic grade, the experience of being bullied, whether they performed regular exercise, and the existence of close friends, were obtained before the program. In addition, the Washington Group Short Set on Functioning [17] was administered to the participants to learn about their disabilities.

*Self-Esteem*

The Japanese versions of the Rosenberg Self-Esteem Scale (RSES) (α = 0.81) [18,19] were employed to assess the self-esteem of the participants before and after the program. The RSES has been standardized, and it is a 10-item self-administered scale that measures self-esteem. Respondents rate their self-esteem on a four-point scale: 1 = strongly disagree, 2 = disagree, 3 = agree, and 4 = strongly agree. A higher score indicates higher self-esteem. The RSES is one of the most useful instruments in the world to assess self-esteem and is widely used in different language versions.

*Mental Health*

Kessler 6-item Psychological Distress Scale-K6 (K6) (AUC Index = 0.94) [20] was used to assess participants’ mental health before and after the program. The K6 is a standardized scale with the 6-item instrument on a 0–4 response set. It is used to assess mental health status. It is widely used as an indicator of the degree of several mental health problems, including psychological stress. The higher the total score, the more serious the mental health condition is likely to be. K6 has been widely employed in different settings in the world.

*Knowledge and Attitude*

Participants were also asked original questions before and after the program on their knowledge and attitude toward diversity based on the current United Nations Human Rights Framework. These questions were developed in collaboration with academic experts on diversity and human rights, public health experts, mental health experts, a United Nations human rights advisor, representatives of organizations of persons with disabilities, and young people. At the end of the questionnaire, questions on existing social support and successful coping strategies were asked to assess positive resiliency. Participants were requested to answer the questionnaire one week before the program and one week after the program.

### 2.3. Procedure

The program was created by a vendor with experience developing exploratory educational programs. This was a pilot diversity and inclusion education program for junior high school students in Japan implemented between December 2021 and February 2022. It involved five 45-min sessions on diversity with a special focus on “pain.” The vendor developed an instruction guide for teachers, and teachers in junior high schools implemented the program based on this guide. The program has an active learning style and is structured as follows:

Session 1: Students share “the pain that I know” among group members and map the pain based on its intensity and commonality. The goal is to understand diversity in the perception of pain among members.

Session 2: Students share what was discussed in each group with the class as a whole and watch a video regarding pains that they did not know about.

Session 3: Students have a group discussion on the value of realizing pains that they did not know about and do homework to find the “invisible pain” (to which they had not paid attention thus far) in their daily life.

Session 4: Each student to select one pain that they want others to know about and think about a message to deliver.

Session 5: Students share the selected pains and messages in groups and reflect on the program.

Before data collection, all procedures and questions were discussed with the school principals and teachers to ensure that the questions were relevant and not intrusive. Informed consent forms were distributed to the parents of the students. The researchers explained that there would be no negative implications even if their children did not participate in the study, and all the data would be stored safely with individual data coded and statistically analyzed as groups. If the parents agreed, they were requested to sign the consent form. Students were also informed that their responses would not affect their academic grades, and teachers would not know their individual answers because all the data would be coded and statistically analyzed.

The procedures for this study were approved by the Institute of Human and Social Sciences Ethics Committee of Kanazawa University (2021-53).

### 2.4. Statistical Analysis

The χ^2^ analysis was used for the comparison of categorical variables, and a Wilcoxon signed-rank sum test was used for continuous variables. In addition, a logistic regression analysis was employed, where whether or not the K6 score worsened after the program (coded 1 if the score worsened after the program, and 0 otherwise) was set as an outcome. The pre-program K6 score and gender, as well as whether the participant has a disability, close friends, and a regular exercise routine, were set as independent variables. Statistical analysis was conducted using SPSS version 28.0 J for Windows (SPSS, Tokyo, Japan). Statistical significance was set at *p* < 0.05.

## 3. Results

Table 1 shows the participants’ sociodemographic data. A total of 18 students (1.8%) answered that they had difficulty seeing, even when wearing glasses; three students (0.4%) had difficulty hearing, even when using a hearing aid(s); four students (0.5%) had difficulty walking or climbing steps; 28 students (3.6%) had difficulty remembering or concentrating; three students (0.4%) had difficulty with self-care, such as washing all over or dressing; and 22 students (2.8%) had difficulty communicating, for example, understanding or being understood. Based on these answers, participants who answered that they have difficulty in one or more of these—60 students (7.7%)—were categorized as the group with disabilities.

Further, 126 students (16.2%) reported that they had experienced being bullied, 398 (51.3%) reported that they exercised regularly, and 609 (78.5%) answered that they had close friends. 

Table 2 shows that for questions related to knowledge and attitude toward diversity and inclusion, the ratio of those who answered correctly increased significantly for most of the questions. However, the ratio of correct answers decreased for some key questions, such as those on the importance of listening to the voices of minorities and of asking about their needs rather than just assuming them.

Table 3 shows that scores of the RSES improved significantly after the program (Median = 16.00, 1st to 3rd percentile = 16.00–17.00) compared to before the program (Median = 15.00, 1st to 3rd percentile =14.00–16.00, W = 63,814.00). The score ranged from 0 to 40, and the difference in average scores was very small at 0.19. For K6, the post-program score (Median = 5.00, 1st to 3rd percentile = 2.00–9.00) was significantly worse than the pre-program score (Median = 4.00, 1st to 3rd percentile = 1.00–8.00, W = 79,916.00). Although not included in the table, analysis among participants with disabilities revealed no significant difference in the paired t-test on K6 scores between the pre- and post-intervention (pre-intervention: M = 9.23, SD = 5.56; post-intervention: M = 10.14, SD = 6.04; *t* =−1.06, *p* = 0.30). However, their pre-intervention K6 scores were almost double that of participants without disabilities (pre-intervention: M = 4.41, SD = 4.17; post-intervention: M = 5.67, SD = 4.62; *t* = −7.71, *p* < 0.01). 

According to the previous literature, social support, personal background, and educational environment may affect the mental health conditions of adolescents. These factors were chosen as independent variables for the linear regression model. Table 4 shows the results of the logistic regression analysis. Lower K6 scores before the program and worse academic grades at school had significantly higher odds ratios (1.13 and 1.28, respectively). Being a girl, not having a disability, and having close friends led to worse K6 scores after the program. 

## 4. Discussion

This study evaluated the impact of a diversity education program on young adolescents by comparing changes in knowledge and attitudes related to diversity, self-esteem, and mental health before and after the program. Regarding knowledge and attitude related to diversity and inclusion, the ratio of correct answers increased from before to after the program for all but two questions. However, the ratio of those who answered correctly remained as low as 20–40% even after the program for about half of the questions. Therefore, this result could not be understood as a meaningful improvement in terms of knowledge and attitude. In addition, in two questions on the importance of listening to the voices of minorities and what is required to understand the feelings and needs of others, the ratio of correct answers decreased. Listening to the voices of marginalized populations and consulting with them are foundational aspects of promoting diversity and inclusion. If the program decreases the number of students who correctly understand the importance of these aspects, the program’s effectiveness in terms of basic knowledge and attitude toward diversity and human rights is doubtful. The current human rights standards recommend listening to the voices of every person to understand their real needs; this is because preferences, contexts, and surrounding environments are different for each person at each time, and decision-making based on stereotypes without consulting the relevant persons could be harmful.

Self-esteem generally improved after the program, although the degree of change was small. However, when we examined students who were susceptible to marginalization, such as students with disabilities, no significant improvement was detected between before and after the program.

Overall, the mental health scores of the students deteriorated after the program. This might be associated with the fact that the program attempted to examine diversity and inclusion through “pain.” The worsened scores after the program do not necessarily mean poor mental health since many of the changes occurred within the non-clinical range, and the long-term sustainability of the deterioration is unknown. However, there is evidence that debriefing in a group immediately after a crisis situation increases the symptoms of posttraumatic stress disorder; therefore, such debriefing is not recommended [21]. Given the age of young adolescents, safety is a critical priority. Accordingly, further analyses to ensure that no harm was done will be necessary.

The development process of this educational program may have affected these results. The development team mostly comprised men who were older than 30 years. Although they had organized intensive consultations with schoolteachers and principals, they did not involve experts on diversity, organizations of persons with disabilities, women, or young people, even though a famous writer who uses a wheelchair engaged in several consultations and appeared in the video material. Moreover, the vendor had only a brief online consultation with a representative of an organization of persons with mental health conditions or psychosocial disabilities upon the request of the organization that hired the vendor. These factors might have negatively affected the program. Further, the results led to the following key lessons:

First, since educational programs involve human interventions, especially when children or young adolescents are the participants, it is important for program developers to involve experts. In this way, the interventions will be based on evidence as well as the outcomes and lessons learned from past studies, including meta-analyses [7,8,11]. The programs should also be based on good practices so that they are effective and not harmful. In this study, it was shown that even though the developer attempted to deliver a good program, it had limited effects on improving the knowledge, attitudes, and self-esteem of participants; appeared to promote undesirable attitudes; and led to worsening mental health at the end of the program. While there is a possibility that the program was developed for different purposes and methodologies, it is still important to examine its effectiveness and the possibility that it could do harm, especially among children, young people, and other marginalized populations. This is critical because various studies have reported that young and marginalized people have a higher risk of mental health conditions including suicide [22]. It should be noted that even similar interventions can result in very different outcomes depending on various factors, including differences in methodologies, delivery orders, age, culture, and gender; therefore, a certain process needs to be followed, as mentioned. 

Today, the importance of key stakeholder participation based on the principle of “nothing about us without us” is increasingly being understood and implemented widely [23]. This principle should also apply when developing educational programs. That is, the program development and implementation processes should involve various people, including persons with disabilities and young people. 

In this study, many students did not provide correct or desirable answers to questions on knowledge and attitude toward diversity, even after completing the program. This could be because the program was developed without consultation with experts, persons with diversity, and young people themselves; therefore, the program did not include the current and dynamic concepts of diversity and disability. For example, the vendor utilized “pain” as a keyword to explore the world of diversity; this approach may have been effective if it had been based on evidence, good practices, and dialogue with persons with disabilities in consideration of the principle “nothing about us without us” adopted in international guidelines regarding disability policies [23]. Naively linking diversity and disability to pain itself could be criticized for being based on the charity model of disability [24], a possibly harmful approach based on a lack of understanding of the social model of disability [4]. This fact clearly indicates that intervention based on evidence, good practices, and dialogue with persons with disabilities is essential when developing an educational program. Moreover, considering that the mental health of this study’s participants worsened, evidence-based and participatory processes are necessary when developing and implementing programs related to diversity and differences that are closely associated with identity and mental health. This is especially important when reaching out to young people who are in the process of developing their identities. Given the age of the participants, there is a chance that they might experience perspective and/or behavioral changes in the long run upon going through such a program. Thus, it is necessary to examine the program’s long-term effects from additional perspectives.

This study has several limitations. The program outcome was evaluated only once, immediately after the program, and the long-term effects are unknown. The evaluation was based on originally developed questions on knowledge and awareness, self-esteem, and mental health. The inclusion of more dimensions and perspectives may be useful. Furthermore, during the study period, while the classes in secondary schools in Japan were conducted normally, there were some COVID-19 restrictions regarding extracurricular activities and traveling across the prefecture. Additionally, fears around the pandemic and the high death rates may also have negatively affected the mental health condition of the participants. Naglkerle R^2^ is low (0.09), and the result and discussion based on logistical regression analysis may have some limitations. In other words, many other factors contribute to the deterioration of mental health among young adolescents, and this study only shows a part of it. Therefore, the interpretation of the results should be cautious. Further empirical studies are desirable.

The findings suggest the importance of scientifically evaluating diversity and inclusion education throughout the development, implementation, and follow-up phases. As diversity, including gender and disability, is closely interlinked with identity, relationships, and mental well-being, education programs can negatively increase stigma, discrimination, and bullying based on social barriers. Efforts to ensure that the education program is based on current knowledge and methods so that it does not deliver incorrect information, increase stigma, and result in harmful practices, must be part of the development process. Diversity-related interventions should include scientific evaluations, mainstreaming the participation of key stakeholders, including persons with disabilities, an evidence- and good practices-based approach, and the “do no harm” and “nothing about us without us” principles as the standard.

## 5. Conclusions

This study evaluated the impact of a diversity education program on young adolescents. The ratio of those who answered the knowledge and attitude questions correctly increased significantly for most questions, while the ratio decreased significantly for two questions. Self-esteem scores improved significantly after the program, but the difference was very small. Mental health became significantly worse after the program. A logistic regression analysis indicated that lower K6 scores before the program and worse academic grades had significantly higher odds ratios. This indicated the importance of developing processes based on evidence and the “do no harm” and “nothing about us without us” principles. 

## Figures and Tables

**Table 1:** Participants’ socio-demographic data.

	City A		City B		City C		City D	
	Frequency ^a^/ M ^b^	% ^a^/ SD ^b^	Frequency ^a^/ M ^b^	% ^a^/ SD ^b^	Frequency ^a^/ M ^b^	% ^a^/ SD ^b^	Frequency ^a^/ M ^b^	% ^a^/ SD ^b^
Age ^b^	13.29	0.59	12.86	0.38	12.82	0.38	12.66	0.48
Gender ^a^								
Men	108	51.40	49	49.50	52	40.30	106	45.10
Women	95	45.30	50	50.50	75	58.10	129	54.90
Others	7	3.30	0	0.00	2	1.60	0	0.00
with disabilities ^a^	21	10.00	5	5.10	12	9.30	22	9.40
Academic grade ^a^								
Very good	10	4.80	7	7.10	4	3.10	10	4.30
Good	24	11.50	20	20.20	23	17.80	38	16.50
Average	75	35.90	36	36.40	45	34.90	86	37.20
Not good	76	36.40	25	25.30	37	28.70	73	31.60
Not very good	25	11.50	11	11.10	20	15.50	24	10.40
Experience of being bullied ^a^	28	13.50	22	22.40	22	17.10	54	23.30
Regular exercise ^a^	108	51.40	56	56.60	83	64.30	151	64.80
Existence of close friends ^a^	188	89.50	89	89.10	119	84.80	213	93.00

^a^ indicates the categorical variable and the frequency of the item and the percentage it accounts for; ^b^ indicates the continuous variable and its mean and SD.

**Table 2:** Comparison of knowledge and attitude related to diversity before and after the program.

Questions (x Indicates Wrong)	Pre or Post	n	Correct Answers		*χ* ^2^	
			n	%		
The social model of disability was adopted at the United Nations	Pre	664	138	20.80	69.40	**
	Post		315	47.20		
Social barriers are the key factors of disability, and we need to address them	Pre	662	247	37.30	47.60	**
	Post	667	399	51.40		
Universal design means design, spaces, and systems that meet the needs of various people, hence can be used by them	Pre	658	526	79.90	178.15	**
	Post	673	567	84.20		
Persons with disabilities should have treatment and surgery so that they can live independently (x)	Pre	662	238	36.00	121.73	**
	Post	669	307	45.90		
Persons with disabilities are vulnerable and people around them should provide help even when the persons do not want help (x)	Pre	661	219	33.10	150.11	**
	Post	669	254	38.00		
Stereotypes and prejudices can be a social barrier	Pre	657	127	19.30	54.48	**
	Post	674	139	20.60		
Minorities with different opinions should be respected with dignity	Pre	656	453	69.10	85.83	**
	Post	672	509	75.70		
The majority rule is most preferable since it is equitable (x)	Pre	656	360	54.90	120.51	**
	Post	673	397	59.00		
Differences among people lead to a better world	Pre	656	428	65.20	178.15	**
	Post	674	518	76.90		
It is important to prioritize the opinions of the majority rather than minority voices or those who are silent (x)	Pre	654	288	44.00	86.73	**
	Post	674	279	41.40		
Others’ feelings can be precisely understood if we imagine them (x)	Pre	656	315	48.00	92.66	**
	Post	674	300	44.50		
Those with different perceptions can become happier if they change and start to follow others (x)	Pre	654	379	58.00	61.69	**
	Post	666	427	64.10		
When deciding something, it is important to involve various stakeholders who are affected by the decision, in addition to leaders and experts	Pre	652	537	82.40	47.27	**
	Post	669	567	84.80		
When we try to support someone, and are not capable enough to respond to their needs, we can link the person to those who can better support them	Pre	651	533	81.90	41.31	**
	Post	668	551	82.50		
When we try to support persons with disabilities, we need to follow specific methods that are defined by disability types (x)	Pre	650	96	14.80	28.33	**
	Post	669	148	22.10		
To realize a better world, it is useful to increase options rather than having only one option	Pre	646	457	70.70	86.86	**
	Post		517	77.30		

** *p* < 0.01

**Table 3:** Comparison of the K6 and Rosenberg Self-Esteem Scale before and after the program.

	Before the Program		After the Program						
	Median	1st to 3rd Percentile	Median	1st to 3rd Percentile	Mean Rank	Sum of Rank	Z	W ^a^	
RSES (n = 602)	15.00	14.00–16.00	16.00	16.00–17.00	200.03	36,005.00	2.04	63,814.00	*
K6 (n = 578)	4.00	1.00–8.00	5.00	2.00–9.00	223.34	51,146.00	3.95	79,916.00	**

** *p* < 0.01, * *p* < 0.05 ^a^ Wilcoxon signed-rank sum test. The Rosenberg Self-Esteem Scale (RSES) shows that higher scores indicate a higher level of self-esteem. The Kessler 6-Item Psychological Distress Scale (K6) shows that higher scores indicate a higher level of stress.

**Table 4:** Logistic regression analysis on K6 score becoming worse or not (n = 575).

Outcome: K6 Becoming Worse or not ^a^			
Independent Variables	OR	95% CI	
Gender ^b^	0.23	0.36–1.39	
Academic grade	1.28	1.08–1.53	**
Whether one has a disability	0.53	0.27–1.07	†
Whether one has close friends	1.89	0.96–3.71	†
Whether one has a regular exercise routine	0.91	0.63–1.30	
K6 score before the program	0.88	0.84–0.92	**

** *p* < 0.01 † *p* < 0.1 Naglkerle R^2^ = 0.09 OR = Odds Ratio; ^a^ coded 1 if the K6 score became worse after the program intervention, 0 for others. ^b^ girl coded 0, boy coded 1, other coded 2.

## Data Availability

Not applicable.
